# Oxygen minimum zone: An important oceanographic habitat for deep‐diving northern elephant seals, *Mirounga angustirostris*

**DOI:** 10.1002/ece3.3202

**Published:** 2017-07-03

**Authors:** Yasuhiko Naito, Daniel P. Costa, Taiki Adachi, Patrick W. Robinson, Sarah H. Peterson, Yoko Mitani, Akinori Takahashi

**Affiliations:** ^1^ National Institute of Polar Research Midori‐cho Tachikawa, Tokyo Japan; ^2^ Long Marine Laboratory Center for Ocean Health Institute of Marine Sciences University of California Santa Cruz CA USA; ^3^ Field Science Center for Northern Biosphere Hokkaido University Bentencho Hakodate, Hokkaido Japan; ^4^ Present address: Department of Biological Sciences Graduate School of Science The University of Tokyo Tokyo 113‐0032 Japan

**Keywords:** bio‐logging, elephant seal, feeding efficiency, jaw‐motion recorder, marine mammal, oxygen minimum zone

## Abstract

Little is known about the foraging behavior of top predators in the deep mesopelagic ocean. Elephant seals dive to the deep biota‐poor oxygen minimum zone (OMZ) (>800 m depth) despite high diving costs in terms of energy and time, but how they successfully forage in the OMZ remains largely unknown. Assessment of their feeding rate is the key to understanding their foraging behavior, but this has been challenging. Here, we assessed the feeding rate of 14 female northern elephant seals determined by jaw motion events (JME) and dive cycle time to examine how feeding rates varied with dive depth, particularly in the OMZ. We also obtained video footage from seal‐mounted videos to understand their feeding in the OMZ. While the diel vertical migration pattern was apparent for most depths of the JME, some very deep dives, beyond the normal diel depth ranges, occurred episodically during daylight hours. The midmesopelagic zone was the main foraging zone for all seals. Larger seals tended to show smaller numbers of JME and lower feeding rates than smaller seals during migration, suggesting that larger seals tended to feed on larger prey to satisfy their metabolic needs. Larger seals also dived frequently to the deep OMZ, possibly because of a greater diving ability than smaller seals, suggesting their dependency on food in the deeper depth zones. Video observations showed that seals encountered the rarely reported ragfish (*Icosteus aenigmaticus*) in the depths of the OMZ, which failed to show an escape response from the seals, suggesting that low oxygen concentrations might reduce prey mobility. Less mobile prey in OMZ would enhance the efficiency of foraging in this zone, especially for large seals that can dive deeper and longer. We suggest that the OMZ plays an important role in structuring the mesopelagic ecosystem and for the survival and evolution of elephant seals.

## 1 INTRODUCTION

The mesopelagic zone, a vast oceanic region typically between depths of 200–1,000 m, is one of the largest ecosystems on earth (Irigoien et al., 2014; Robinson, Steinberg, et al., 2010; Robison, 2004) and forms a critical foraging habitat for a variety of large predators, that is, fishes, turtles, penguins, toothed whales, and seals (Block et al., 2011; Charrassin et al., 2001; Cherel, Ducatez, Fontaine, Richard, & Guinet, 2008; Eckert, Eckert, Ponganis, & Kooyman, 1989; Miller, Johnson, & Tyack, 2004; Potier et al., 2007).

While many large predators depend on mesopelagic prey, it is apparent that the biota of this zone is strongly affected by the amount of dissolved oxygen which varies largely according to depth (Robison, 2004). The shallower part of the mesopelagic zone is rich in biota, but the deeper part is biota‐poor as an oxygen minimum zone (OMZ) generally develops forming a habitat‐restrictive anoxia zone (Robinson, Steinberg, et al., 2010; Robison, 2004). Recent upward expansion of the OMZ may threaten the lives of large predators by reducing their biota‐rich foraging zone (Bograd et al., 2008; Stramma et al., 2008, 2012), but the importance of the mesopelagic zone, including the deep anoxia zone, as a foraging habitat for large predators has not been fully described.

Despite the low prey availability and high costs of diving to great depths in terms of energy and time, various marine mammals are known to make deep dives and may use the OMZ as their predominant foraging habitat, which appears to contradict the predictions from optimum foraging theory (Stephens & Krebs, 1986). Investigation of foraging success is key to understanding their puzzling foraging behavior and the ecological role of the OMZ. Yet, measuring foraging success based on gain and cost ratios (feeding efficiency, i.e., gain per unit of energy or time) in the mesopelagic zone is extremely difficult, and has thus far prevented an understanding of how deep divers maintain foraging success in the OMZ when challenged with the increased costs of deep diving. Hence, the role of marine mammals in the deep‐sea ecosystem remains largely unknown (Robison, 2004).

Elephant seals are typical mesopelagic foragers and dive continuously for 2–3 months during the postbreeding migration (Bailleul et al., 2007; Guinet et al., 2014; Hindell, Bradshaw, Sumner, Michael, & Burton, 2003; Le Boeuf et al., 2000; Robinson, Simmons, Crocker, & Costa, 2010). They dive throughout the day and night forming an apparent diel pattern in the dive depths and perform very deep dives episodically during daytime possibly to the OMZ. These deep dives consistently exceed depths of 800 m, with a maximum observed dive depth of 1,735 and 2,133 m in northern and southern elephant seals, respectively (McIntyre et al., 2010; Robinson et al., 2012). In the past several decades, foraging studies on seals have relied on various approaches including stomach temperature (Kuhn, Crocker, Tremblay, & Costa, 2009), dive profile and swim speed change (Crocker, Le Boeuf, & Costa, 1997; Hindell, Slip, & Burton, 1991; Le Boeuf, Costa, Huntley, & Feldkamp, 1988; Thums, Bradshaw, & Hindell, 2011), movement pattern (Le Boeuf et al., 2000; Robinson, Simmons, et al., 2010), body density (buoyancy) change (Biuw et al., 2007; Robinson et al. 2010), and video (Davis, Fuiman, Williams, Horning, & Hagey, 2003). However, all these techniques lack the temporal and spatial resolution and/or coverage to estimate foraging success precisely for long periods, and this has limited our understanding of how animals forage in the deep sea.

A recently developed method to observe the feeding attempts of marine mammals using accelerometers on the jaw or the head of seals has made it possible to estimate putative feeding rates (hereafter, feeding rates) dive by dive covering the entire period of migration (Adachi et al., 2017; Jouma'a et al., 2015; Naito et al., 2013; Richard, Cox, Picard, Vacquié‐Garcia, & Guinet, 2016). These results have raised the hypotheses that elephant seals forage on small mesopelagic prey (10–20 g) on average during migration (Guinet et al., 2014; Naito et al., 2013), which accord with stable isotope studies (Cherel et al., 2008), and that they fine‐tune their diving behavior by adjusting their swimming effort in relation to prey patch depth and density and according to changes in buoyancy (Adachi et al., 2014; Jouma'a et al., 2015; Richard et al., 2016). Despite these recent studies, the entirety of their dietary choice, characterized within the mesopelagic zone and particularly in the OMZ, remains largely unknown. Although these methods are still unable to estimate the size of each prey ingested, it allows us to estimate their foraging success comparatively against cost (i.e., dive cycle time in this study) or to examine variation in feeding rates among individuals during the entire period of migration. Elephant seals can be used to gain insights into the importance of certain prey in the OMZ with implications for other deep‐diving marine megafauna.

Here, we aimed to reveal how female northern elephant seals manage their foraging success in the deep mesopelagic zone, particularly in the biota‐poor OMZ, despite the high cost in time of deep diving during the limited period of their postbreeding migration. For this purpose, we monitored the variability in the seal's feeding rates based on jaw movement events (JME) using jaw motion recorders (Naito et al., 2013) attached to 14 seals. We analyzed their feeding rates in relation to the body mass and dive depths of the seals. These results were supplemented with a video recording from one individual obtained using a new video logger triggered by depth and the seal's head motion. Based on these results, we discuss the importance of the OMZ as a foraging habitat for elephant seals and other deep‐diving marine mammals.

## 2 MATERIALS AND METHODS

### 2.1 Field experiments

We deployed jaw motion recorders on the left, lower mandible of 15 seals (three in 2011, four in 2012, and eight in 2013, respectively). We wrapped the recorders in rubber splicing tape and attached to high‐tension mesh netting with cable ties, and then glued to the pelage using epoxy resin. All seals were fitted with 0.5 W Argos transmitters (Wildlife Computers, Redmond, WA, USA) on their head for tracking the migration paths, and VHF transmitters (ATS, Isanti, MN, USA) on their back for locating seals upon return to the colony site at the end of the foraging migration. In addition, two video units were used in this study, which were triggered to start recording by a three‐way onboard system that included the start time, start depth, and the first head‐strike motion after the set time and depth. In this study, one video was programmed for shallow depths (>400 m) and the other for deep depths (>800 m), with start times of 3 weeks from deployment (Table 1). Four seals carried swim stroke recorders (Adachi et al., 2014) on their back, but the results are not used here.

**Table 1:** Summary of morphometrics and foraging behavior of northern elephant seals in this study

Seal ID	Days at sea	Body mass at departure (kg)	Body mass at arrival (kg)	Mean body mass (kg)	Body mass gain (kg)	Mean standard body length (cm)	Total no. of JME	No. of JME/day	No. of foraging dives	No. of none and miscellaneous foraging dives	Loggers
1015	73.4	319.0	389.0	354.0	70.0	262.5	167,868	2,287.04	4,178	435	JMER, SSR
3236	72.2	308.2	Not scaled			256.5	123,027	1,705.01	4,264	586	JMER
T35	74.6	340.7	426.7	383.7	86.0	263.5	94,599	1,267.71	3,227	549	JMER, SSR
2024	81.1	297.2	396.9	347.1	99.7	253.0	107,861	1,329.63	4,017	950	JMER
R382	68.4	432.3	499.5	465.9	67.1	291.5	69,715	1,019.67	2,988	757	JMER, SSR
U239	69.3	331.5	408.1	369.8	76.6	262.0	136,665	1,970.74	3,359	612	JMER
U954	70.8	256.0	337.0	296.5	81.0	242.5	136,983	1,934.61	3,632	692	JMER, SSR
18129	75.4	345.8	468.0	406.9	122.2	266.5	126,113	1,672.78	3,757	803	JMER
U256	60.6	357.0	432.8	394.9	75.8	273.0	90,836	1,499.36	2,523	865	JMER
U754	65.7	332.7	402.5	367.6	69.9	265.0	90,836	1,382.53	3,176	332	JMER
U848	80.1	352.0	436.8	394.4	84.8	274.0	100,135	1,249.38	3,401	771	JMER
X334	66.9	302.0	Not scaled			262.5	106,125	1,586.88	2,998	1,133	JMER
X349	79.0	314.1	369.3	341.7	55.2	257.0	119,226	1,509.64	3,546	1,118	JMER
X387	72.5	307.1	391.5	349.3	84.3	267.0	101,769	1,404.34	3,496	840	JMER, VR^a^
2161	45.7	458.9	502.8	480.9	43.9	288.0					JMER^b^, VR
Avg	70.4	337.0	420.1	381.0	78.2	265.6	112,268	1,559	3,469	746	

JMER, jaw motion event recorder; SSR, swim stroke recorder; VR, video recorder.

a Unsuccessful.

b Malfunction.

Fieldwork was conducted at the Año Nuevo State Reserve, California, USA, in February of 2011–2013. We used an intramuscular injection of Telazol (Tiletamine hydrochloride and Zolazepam hydrochloride, Fort Dodge Animal Health, Fort Dodge, IA, USA) to chemically immobilize seals for attachment of the recorders. Body mass and other morphometric measurements were obtained using standard protocols (Le Boeuf et al., 2000; Robinson, Simmons, et al., 2010) at instrument deployment and recovery before leaving the colony and after arriving back at the colony. We could not measure the body mass for one seal at arrival time (seal ID: ×387). Thus, we estimated the body mass using the body mass loss rate on land, and time between weighing and arrival time determined by a VHF transmitter on the animal's back. We were also unable to measure the body mass of two seals when they returned, because irregular rocky topography prevented the measurement of body mass.

### 2.2 Instruments

We developed a long‐term jaw‐motion recorder (KKL; “Kami Kami Logger,” Little Leonardo Co., Tokyo, Japan; diameter 20.2 mm, length 73 mm, mass 48 g: (Naito et al., 2013)). To extend the recording duration with a high‐speed acceleration sampling rate, an onboard data‐processing algorithm was developed to detect JME, which were identified using an amplitude threshold for detection of the events, and the loggers stored the number of events counted every five‐seconds. In parallel with these records, the instruments recorded dive depth (range: 0–2,000 m with an accuracy of 10 m) and temperature every five‐seconds (range: −20–50 °C).

To identify prey types, we developed a new specialized video system for the efficient monitoring of prey in the deep sea (acceleration‐triggered video system: ATVS; “Kami Kami video”, Little Leonardo Co., Tokyo, Japan). The acceleration sensor was particularly important to detect the head‐strike motion that triggered the ATVS to start recording prey in a patch efficiently. We used two 850‐nm LEDs for the light source (SFH4232, OSRAM, Regensburg, Germany), which allowed objects to be visible within 60 cm from the mouth of a seal. The camera and light unit were strengthened to withstand water pressure up to 3,000 m. The dimensions of the ATVS were 28 mm in diameter both for video and strobe, and a length of 148 and 128 mm for the video and strobe, respectively. Weights were 156 and 126 g for the video and strobe, respectively.

### 2.3 Data analysis

We obtained a complete data set of JME and diving behavior from 14 seals over the entire postbreeding trip. Although the quantity of video footage was limited, we obtained prey images from the video programmed to work at depths of around 800 m, which were used in this study for identification of prey in the OMZ. For the analysis of behavioral data, we used the mask function of the Ethographer software package (Sakamoto et al., 2009) and Igor Pro software (v6.03; WaveMetrics, Lake Oswego, OR, USA).

Metrics of foraging behavior began with the first foraging dive and ended with the last foraging dive, which were several hours shorter than the trip length determined by the first and last dive. The start and end time of each dive, dive duration, and surface time were based on the time when seals reached and returned to a depth of 10 m.

We estimated the time budget of the seal's continuous diving behavior that lasted from the start to the end of the foraging migration. We categorized their dives into three types using a frequency distribution of the number of JME for each dive (i.e., foraging dive: number of JME ≧ 5, nonforaging dive: number of JME = 0, miscellaneous dive: number of JME = 1–4). We also separated surface time >300 s as extended surface intervals, which was determined visually from a frequency distribution of the surface time for each seal. Argos data obtained from the seals were processed using a speed and turn angle filter and then smoothed using a state‐space model (Robinson et al., 2012).

### 2.4 Feeding rates for comparison

In this study, we examined variability in JME‐based feeding rates using the following biological assumptions: (1) Feeding rates will increase with body mass to serve larger metabolic demands (Boyd, 2002; Costa, 1993), if all seals forage on similar mesopelagic small prey on average based on the reports on numerous prey catch attempts by suction feeding mode (Jouma'a et al., 2015; Naito et al., 2013; Richard et al., 2016) and myctophids as predominant prey as shown by stable isotope analyses (Bailleul et al., 2010; Cherel et al., 2008) and by head‐mounted camera (Naito et al., 2013); (2) feeding rates need to be enhanced in all seals to counteract increasing diving cost in very deep V‐shaped dives to the OMZ to maintain a positive energy balance (depth effect assumption; Stephens & Krebs, 1986).

The body mass of elephant seals is variable during migration and accordingly their prey requirements may vary with changing body mass. We used mean body mass (average of start and end body mass) to examine the effect of body mass on feeding rates during migration. As the seals spent time on shore after the deployment and/or before the recovery of instruments, corrections were necessary to estimate mass at departure and arrival. We corrected our departure and arrival body mass data based on equations derived from serial mass measurements of fasting female seals from previous studies (mass change [kg day^−1^] = 0.51 + 0.0076·mass, *n* = 27, *r* ^2^ = .79, *p* < .01; D.Crocker & D.Costa unpubl. in Simmons et al., 2010).

### 2.5 Oxygen concentration and water temperature data

We obtained dissolved oxygen and water temperature data from the NOAA data center (NOAA World Ocean Data Center, averaged with values collected from 1955 to 2012, http://data.nodc.noaa.gov/woa/WOA13/DOC/woa13v2). We also obtained dissolved oxygen concentrations and water temperature profiles (NOAA World Ocean Data Center, representing the average of one‐degree‐square at 44.5°N, 130°E).

### 2.6 Statistical analysis

Statistical analyses were carried out in the statistical program R (v. 2.15.3, Foundation for Statistical Computing, Vienna, Austria). The *lm* function in the *stats* package was used to fit linear models (LM). The *lmer* function in the *lme4* package was used to fit generalized linear mixed models (GLMMs) that included the individual as a random effect. In all LMs and GLMMs, we calculated the Akaike information criterion corrected for small sample size (AICc) to select the best model (the model with lowest AICc). We also calculated the difference in AICc value (ΔAICc) of a candidate model from the model with lowest AICc, and considered the models with ΔAICc < 2 to have marginal support and ΔAICc > 2 to have no support. Then, the *R* ^2^ or marginal *R* ^2^ values were calculated to evaluate the variance explained by fixed effects, providing a goodness of fit of each LM and GLMM, respectively. Data are presented as means ± standard deviation (*SD*) unless otherwise stated.

## 3 RESULTS

### 3.1 Data set and diving costs

**Figure 1:**
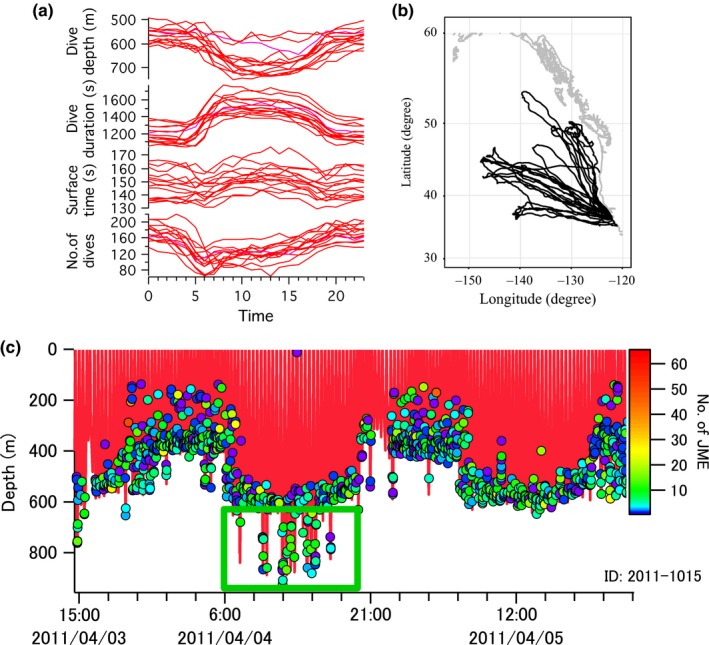
(a) Diel pattern in the dive behavior of 14 seals. Although variation was observed among individuals, all seals adjusted their diving behavior according to the vertical diel migration of prey (average value calculated every hour). (b) Migration paths of the 14 postbreeding adult female seals in the Northeast Pacific. All seals migrated within the normal range of postbreeding female northern elephant seals. (c) Extracted example of dive depth and nJME. Distribution pattern of dive depth (red line) and the depths where jaw motion events (JME) occurred (circle) showed an apparent diel pattern. Seals showed very deep diving episodically during daytime hours (i.e., dives in the green box), suggesting that seals targeted different prey available at deep depths only during daytime

We successfully obtained a complete data set of JME and diving behavior from 14 seals over the entire postbreeding trip (Table 1). Foraging dives were the predominant dive type, ranging from 73% to 91% (including surface time) of the total migration time. Dive type, assigned by number of JME, coupled with the normal diel diving pattern and normal migration pattern of the 14 seals allowed us to estimate feeding rates (Figure 1a,b).

**Figure 2:**
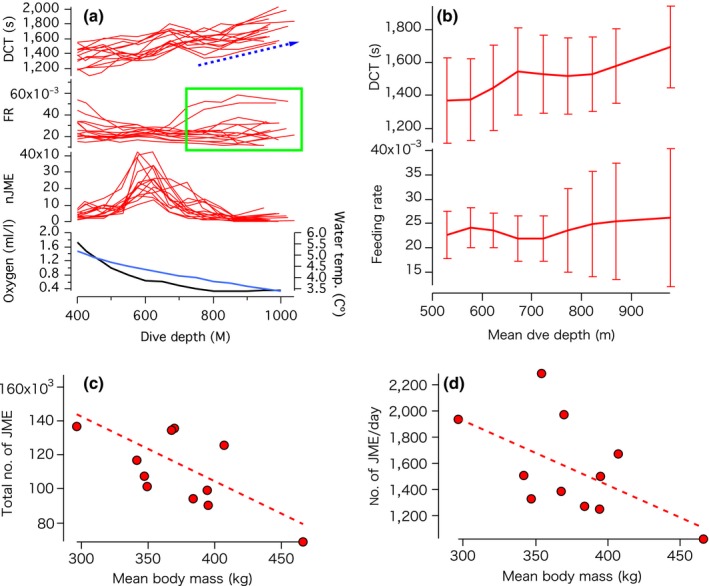
(a) Relationship between dive depth and mean dive cycle time (DCT) (upper), mean feeding rate (FR, which was calculated as nJME/DCT) (upper middle), total number of jaw motion events (JME) (lower middle), and dissolved oxygen concentration (black line) and temperature (blue line) (bottom). Mean DCT and mean FR were calculated for each of 14 seals (shown in different lines) for every 50‐m depth range for the dives with the maximum depth between 400 m and 1100 m (400 m = 400–450 m, 450 m = 450–500 m, etc.). The blue arrow indicates the general trends in DCT in the >800 m depth zone. The green box indicates the irregular zone of FR, where a minimum level is maintained despite the increased DCT. Dissolved oxygen concentration reached the minimum level at 800 m depth (dissolved oxygen and water temperature data: NOAA World Ocean Data Center, representing average profiles at one‐degree‐square at 44.5°N, 130°E). (b) Changes in the mean DCT (upper) and mean feeding rates (lower) in relation to dive depths. Mean values for 14 seals are shown with standard deviations. (c,d) Relationship between the total number of JME during migration and body mass (c) and relationship between nJME/day and body mass (d). Body mass represents mean body mass between body mass at the start and the end of migration. Dashed lines show the regression line of models supported with ΔAICc value <2. Statistical details are shown in Table 2

A total of 48,562 foraging dives and 1,603,476 JME were recorded during postbreeding migrations. While the diel pattern in the depths of JME was apparent from the start to end of migration, very deep dives, beyond the normal or regular diel depth ranges of dives, occurred episodically during daylight hours (green box in Figure 1c). Most JME of all seals during migration appeared in the midmesopelagic zone (500–600 m) and decreased in the 700 and 800 m depth zones suggesting that the 500–600 m depth zone was the main foraging depth zone for all seals (Figure 2a). Dive cycle time (DCT) gradually increased with dive depth as we had assumed (Figure 2a,b; Table 2).

**Table 2:** Summary of generalized linear mixed models (GLMMs) and linear models (LMs). The results of generalized linear mixed models (GLMMs) and linear models (LMs). (a)(b): GLMM with each response variable and the explanatory variable shown in Figure 2b. GLMM includes individual as a random effect. (c)–(g): LM with each response variable and the explanatory variable shown in Figures 2c,d and 3a–c. In all GLMMs and LMs, Akaike's information criterion corrected for small samples (AICc), intercept, and slope coefficient are shown for each model. Also, marginal *R* ^2^ and *R* ^2^ are shown in each GLMM and LM, respectively. The models with the lower AICc are shown in bold (where ΔAICc > 2) or italic type (where ΔAICc <2)

Candidate GLMM	AICc	Intercept	Slope coefficient	Marginal *R* ^2^	
(a)					Figure** ** 2b
**DCT ~ Dive depth**	**1,510.5**	**1,093.5**	**0.65**	**.34**	
DCT ~ 1	1,598.7	1,563.7			
(b)					
**Feeding rate ~ Dive depth**	**−876.0**	**0.018**	**0.0000093**	**.02**	
Feeding rate ~ 1	**−**873.5	0.024			

Candidate LM	AICc	Intercept	Slope coefficient	*R* ^2^	
(c)					Figure** ** 2c,d
*Total no. of JME ~ Body mass*	*281.7*	*250,265.9*	**−** *371.3*	*.34*	
Total no. of JME ~ 1	283.0	111,884.0			
(d)					
*nJME/day ~ Body mass*	*181.1*	*3,377.00*	**−** *4.80*	*.27*	
nJME/day ~ 1	181.3	1,587.80			
(e)					Figure** ** 3a–c
No. dives500 ~ Body mass	176.0	1,521.06	**−**1.36	.04	
**No. dives500 ~ 1**	**172.9**	**1,016.00**			
**No. dives600 ~ Body mass**	**172.6**	**2,971.70**	**−5.02**	**.45**	
No. dives600 ~ 1	176.2	1,100.50			
No. dives700 ~ Body mass	183.2	1,378.68	**−**2.33	.07	
**No. dives700 ~ 1**	**180.4**	**510.50**			
**No. dives800 ~ Body mass**	**163.5**	**−1,182.39**	**3.85**	**.51**	
No. dives800 ~ 1	168.4	251.60			
(f)					
Ratio500 ~ Body mass	**−**19.7	0.2506	0.0001	<.01	
**Ratio500 ~ 1**	**−23.3**	**0.2967**			
Ratio600 ~ Body mass	**−**22.3	0.6610	**−**0.0009	.23	
*Ratio600 ~ 1*	**−** *22.8*	*0.3254*			
Ratio700 ~ Body mass	**−**14.7	0.3516	**−**0.0006	.06	
**Ratio700 ~ 1**	**−17.7**	**0.1427**			
**Ratio800 ~ Body mass**	**−36.9**	**−0.3344**	**0.0011**	**.60**	
Ratio800 ~ 1	**−**29.6	0.0715			
(g)					
*nJME‐based FR500 ~ Body mass*	**−** *91.2*	*0.04829*	**−** *0.00007*	*.33*	
nJME‐based FR500 ~ 1	**−**90.0	0.02382			
**nJME‐based FR600 ~ Body mass**	**−98.8**	**0.04612**	**−0.00006**	**.46**	
nJME‐based FR600 ~ 1	**−**95.0	0.02267			
nJME‐based FR700 ~ Body mass	**−**70.4	0.02962	**−**0.00002	.01	
**nJME‐based FR700 ~ 1**	**−74.0**	**0.02271**			
nJME‐based FR800 ~ Body mass	**−**66.5	0.03749	**−**0.00004	.02	
**nJME‐based FR800 ~ 1**	**−69.9**	**0.02328**	** **	** **	

### 3.2 Feeding rates and body mass

While DCT increased gradually with depth as predicted, feeding rates generally decreased toward 500 m depth but converged between 500 and 600 m, and then slightly increased at 700–800 m and >800 m depths, showing large variations (Figure 2a,b; Table 2). Feeding rates varied largely in the 700–800 m and >800 m depth zones (Figure 2b). Dissolve oxygen concentrations gradually decreased and reached to close to minimum at depths of 700 m and to the minimum at 800 m depths (Figure 2a). Thus, we defined the >800 m depth zone as the OMZ in this study.

According to our assumption that seals fed on homogenous, small prey types (all seals forage on small prey on average (10–20 g; Naito et al., 2013)), and similar feeding rates in each seal were expected. However, we found large variations in feeding rates at 700–800 m and >800 m among the seals (Figure 2a,b). We then examined the effect of seal body mass on the number of JME and feeding rates to test whether larger seals had a higher number of JME than smaller seals to meet higher metabolic needs. Contrary to our prediction, the total number of JME (hereafter nJME) during migration and feeding rates (nJME/day) during migration did not increase with body mass but rather tended to decrease with body mass among the 12 seals (Figure 2c,d; Table 2). This suggests that larger seals might have foraged on larger prey on average to meet their larger metabolic demands.

**Figure 3:**
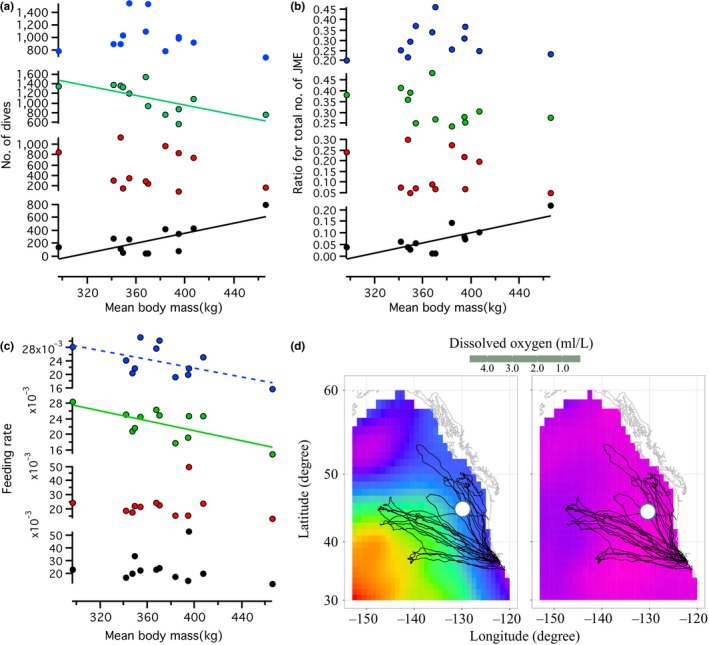
(a–c) Relationship between mean body mass and number of dives (a), nJME ratio for total nJME (b) and feeding rate (FR) (c) at different depth zones. Blue dots, green dots, red dots, and black dots represent 500 m, 600 m, 700 m, and over 800 m depth zones, respectively (averaged data for 500–600, 600–700, 700–800, >800 m depth ranges). Regression lines are shown for the models supported statistically (solid and dashed lines: models supported with ΔAICc > 2 and <2, respectively). Statistical details are shown in Table 2. (d) Averaged dissolved oxygen concentration (ml/L) in spring season in one‐degree‐square resolution at 400 m depth (left) and 800 m depth (right) in the Northeast Pacific (Data: NOAA World Ocean Data Center, averaged with values collected from 1955 to 2012, as described below, http://data.nodc.noaa.gov/woa/ WOA13/DOC/woa13v2). An oxygen minimum zone at 800 m depth prevailed over most of the postbreeding migration region of the seals. The white mark represents the location of video records

To examine the effect of body mass on feeding rates in different depth zones, we compared the relationships between body mass and dive number, the ratio of the number of JME to the total number of JME, and feeding rates in 500–600 m, 600–700 m, 700–800 m and >800 m depth zones. The number of dives decreased linearly with body mass in the 600–700 m depth zones and increased in the >800 m depth zones, while the ratio of the number of JME to the total number of JME increased only in the >800 m depth zones (Figure 3a,b, Table 2). Feeding rates tended to decrease with body mass in 500–600 m and 600–700 m depth zones, but not in 700–800 m and >800 m depth zones (Figure 3c, Table 2). These results suggest that smaller seals tended to dive and feed in shallower depth zones, and larger seals tended to depend more on the >800 m zone compared with smaller seals.

### 3.3 Video observations

**Figure 4:**
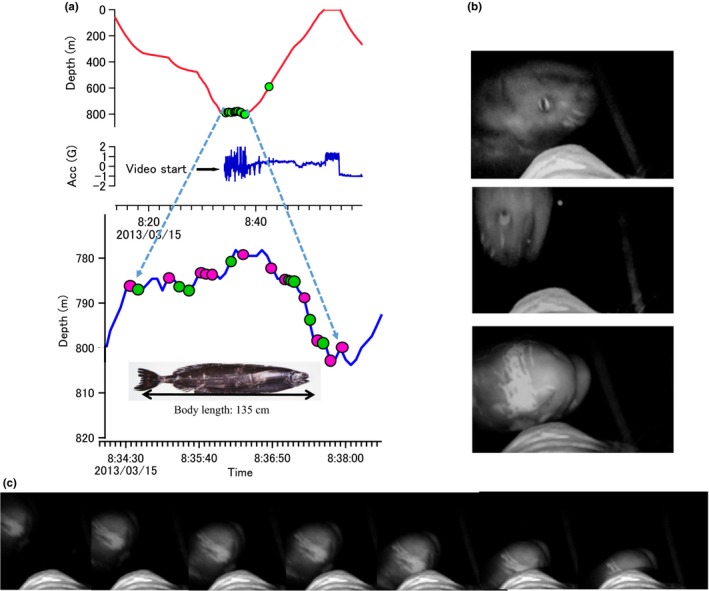
(a) Dive profile with occurrence of jaw motion events (JME; green dots) and the start of video recording. The video recorder recorded for approximately 5 minutes from 08:34:00 on 15 March 2013 (latitude: 44.7050, longitude: −130.3989, sea floor depth: 2200 m). Colored dots on the enlarged dive profile indicate how the seal responded to the fish: captured (green) and captures not confirmed (red). Because of the narrow angle of video vision (about 25 degrees) and the weak light source, only part of the fish appeared in the video footage, but the fish were identified as sections of ragfish by comparing body morphological features with an adult ragfish specimen (inset picture; body length 135 cm) preserved at Kanagawa Prefectural Museum of Natural History which was caught in a set net (depth: 120 m) off the coast of Hayakawa near Odawara City, Kanagawa, Japan, in 1999. (b) Posture of fish immediately before the seal's attack. Fish appeared in the video footage in a variety of postures, for example, dorsal side down (upper image), dorsal side up downward, and lying on the side (lower image). (c) Sequence of video frames right before a seal attack (7 frames). The fish did not show any escape response, keeping its dorsal side down posture until right before the seal attack, suggesting that fish had limited reactivity in the OMZ

The video recorder on a seal (ID: 2013‐2161, programmed to start recording at 800 m) recorded video footages with 21 observations of fish, but only part of the body of fish was visible in the footage due to the limited near‐infrared strobe light range (Figure 4a). These partial images of fish were identified as various body parts of ragfish (*Icosteus aenigmaticus*), by comparing the images with the morphological characteristics of an adult ragfish specimen (e.g., skin ridges on the caudal, lateral and anterior dorsal surfaces, massive lower jaw, skin pattern of the frontal and large nostrils on the round‐shaped upper mandible) (Figure 4a). Of the 21 cases of fish observations, the same individual fish appeared 3–4 times, but the others (17–18 cases) were identified as different individuals. Seals appeared to ingest at least nine of the 17–18 individual fish. It was difficult to confirm the ingestion of fish because the long snout of the seal prevented a view of the mouth area in the video footage. However, we considered that the seal attacked and ingested these small fish, based on distinguishing head movements. One fish appeared 3–4 times over several frames in a short interval without being consumed by the seal. All fish were identified as subadult or adult ragfish based on their skin pattern, which differs from that of juveniles (Allen, 2001). The posture of ragfish was variable (Figure 4b); however, all fish except very large fish kept their posture until the final moment of the seal's attack (Figure 4c).

## 4 DISCUSSION

### 4.1 Diving cost to the OMZ

Our results suggest that, as predicted, the time costs of diving to the OMZ were high as shown by increasing DCT with dive depth (Figure 2b, Table 2). DCT generally increased with depth with some variations among individual seals, but DCT was not a simple function of dive depth and might vary if seals search for prey horizontally at the bottom of dives. Seals appeared to shift their dive time for searching for prey from horizontal to vertical dimensions in deep dives (>700–800 m), which resulted in the changes in dive profiles from a zigzag bottom profile to a V‐shaped profile (Figure 2a). This suggests that seals allocated more DCT for vertical prey searches in deep dives, which resulted in shorter bottom times than in shallow dives. Such V‐shaped dive profiles (Naito et al., 2013) characterized the dives to the OMZ as well as prey distribution pattern in this zone.

### 4.2 Body mass effect and the size of prey

Our study, which aimed to reveal how marine mammals forage efficiently in the biota‐poor OMZ, depended on the accuracy of measurements of energy gain by the jaw motion recorder, that is, accuracy in the number of JME as a comparative index of the amount of prey consumed by the seals. From our previous study, we predicted that JME was a reliable index of digested prey mass based on the following empirical reasons related to the narrow prey size distribution: (1) They use a suction feeding mode functional for feeding on small prey (Bloodworth & Marshall, 2005; Marshall, Kovacs, & Lydersen, 2008; Suzuki, Naito, Folkow, Miyazaki, & Blix, 2009); (2) their morphologically degenerate molar teeth are less functional for feeding on large prey (Abbott & Verstraete, 2005); (3) small fish, that is, micronekton, are dominant in the mesopelagic zone (Cherel et al., 2008; Irigoien et al., 2014; Naito et al., 2013; Robinson, Steinberg, et al., 2010; Robison, 2004); and (4) the body size distribution of the dominant animals captured by mesopelagic trawling in the 400–800 m depth in the same area of the Northeast Pacific is very small (Saijo et al., 2017).

Contrary to our prediction, larger seals tended to show lower total numbers of JME and lower average feeding rates during the postbreeding migration (Figure 2c,d, Table 2). If larger seals have larger overall energy requirements than smaller seals (Costa, 1993; Boyd, 2002), this suggests that the size of consumed prey might not be the same for all seals and might be larger for large seals to meet their higher energy requirements. Information on the size distribution of micronekton or nektonic animals in the mesopelagic zone is very limited due to the difficulty of sampling by nets in the deep mesopelagic zone, particularly at depths deeper than 600 m (Benoit‐Bird, Southall, & Moline, 2016; Irigoien et al., 2014; Robinson, Steinberg, et al., 2010; Robison, 2004). Larger seals tended to show lower feeding rates in the 500–600 m and 600–700 m depth zones, but not in 700–800 m and >800 m depth zones (Figure 3c). This may indicate that larger seals generally foraged on larger prey compared with smaller seals in these zones. Large variations in feeding rates among individual seals in the OMZ are difficult to explain (Figure 2a,b). It may simply imply randomness in the size and density distribution of patchy prey in the OMZ. However, diving ability may strongly relate to the efficiency of foraging in this zone, as larger seals showed higher diving frequency and higher feeding ratios in this zone than smaller seals, possibly due to the greater diving ability of larger seals (Halsey, Butler, & Blackburn, 2006; Hassrick et al., 2010; Schreer & Kovacs, 1997; Weise, Harvey, & Costa, 2010). Given the large fish that appeared in video footage and the high predation pressure from seals, we hypothesize that large prey is likely to use the OMZ, where they can slow their metabolism and rest, to escape from predation in the oxygen‐rich upper mesopelagic zone (Childress & Seibel, 1998; Seibel, 2011).

We note that the relationship between body mass and nJME‐based feeding rate is statistically weak due to limited sample size, and our results need to be confirmed by further studies with a larger sample size. In addition, our data may include errors caused by different body mass and metabolic demands at different growth stages or ages (Deutsch, Crocker, Costa, & Le Boeuf, 1994) that may affect nJME‐based feeding rates nonlinearly, even though all the animals in this study were sexually mature.

### 4.3 Prey behavior in the OMZ

We examined how prey type and prey behavior related to the OMZ with a video recorder. Our video recorded the rarely reported or captured ragfish (Allen, 2001). The video records indicated that ragfish were found in patchy locations. Compared to Weddell seals, *Leptonychotes weddellii*, (Naito et al, 2010), elephant seals may be less adept at processing very large prey items at deep depth. This may explain why the seal in our study carrying the video recorder attacked 12 fish, but swam past others. Our video showed the static postures of ragfish until the final moment of the seal's attack, despite their well‐developed caudal fin, which suggested that they were in a state of metabolic suppression, a type of adaptation of fish during the day in the OMZ (Childress & Seibel, 1998; Seibel, 2011), a zone which occurs across most of the migration area of northern elephant seals (0.5 ml/L, Figures 2a, 3d, 4b,c). This video observation suggests that the low oxygen concentrations of the OMZ might make prey animals metabolically immobilized and provide great feeding advantages to all predators but particularly those larger predators that can dive deeper and longer.

### 4.4 Foraging in the OMZ and the midmesopelagic zone

Many toothed whales target large prey, for example, squid, exclusively in the deep seas exceeding 800 m in depth using remote echolocation systems (Clark, 1996; Johnson, Madsen, Zimmer, Aguilar de Soto, & Tyack, 2006; Madsen, Wilson, Johnson, & Hanlon, 2007; Ruiz‐Cooley, Gendron, Aguíñiga, Mesnick, & Carriquiry, 2004; Tyack, Johnson, Aguilar de Soto, Sturlese, & Madsen, 2006; Watwood, Miller, Johnson, Madsen, & Tyack, 2006). It is also reported that their prey showed a largely heterogeneous horizontal distribution at depth (Benoit‐Bird et al., 2016), which is similar to the episodic patchy prey distribution of elephant seals in the OMZ. Whereas heterogeneous horizontal distribution is obvious in the OMZ, midmesopelagic small prey animals are distributed in a homogeneous pattern which is related to the continuous diving pattern by elephant seals (Le Boeuf et al., 2000; Naito et al., 2013; Robinson, Simmons, et al., 2010). This continuous diving pattern is completely different from that of toothed whales that dive less frequently to deeper depths compared with elephant seals. This comparison suggests that foraging on heterogeneously distributed prey in deep seas needs a remote prey sensing system, such as the echolocation of toothed whales. In this context, we suggest that an investigation of how elephant seals detect prey in the OMZ remotely is another key to understanding their foraging in this zone, as efficiency in prey detection in the OMZ ultimately determines their foraging success. Here, we suggest that the OMZ plays an important role not only in structuring the mesopelagic ecosystem but also for the survival and evolution of deep‐diving marine mammals.

## AUTHOR CONTRIBUTIONS

Y.N., A.T., and D.P.C. designed research; P.W.R. and S.H.P. led and performed fieldwork with Y.N., D.P.C., T.A., and A.T.; T.A. conducted statistical analysis; Y.M. provided trawl net survey information; Y.N. wrote the manuscript with input from other authors.

## DATA ACCESSIBILITY

Data used in this paper are available upon request to Akinori Takahashi at National Institute of Polar Research.

## CONFLICT OF INTEREST

None declared.
